# Lithium’s antiviral effects: a potential drug for CoViD-19 disease?

**DOI:** 10.1186/s40345-020-00191-4

**Published:** 2020-05-20

**Authors:** Andrea Murru, Mirko Manchia, Tomas Hajek, René E. Nielsen, Janusz K. Rybakowski, Gabriele Sani, Thomas G. Schulze, Leonardo Tondo, Michael Bauer, for the International Group for The Study of Lithium Treated Patients (IGSLi)

**Affiliations:** 1 Bipolar and Depressive Disorders Unit, IDIBAPS CIBERSAM, Hospital Clinic, Barcelona, Catalonia Spain; 2 Section of Psychiatry, Department of Medical Sciences and Public Health, University of Cagliari, Cagliari, Italy; 3 Unit of Clinical Psychiatry, University Hospital Agency of Cagliari, Cagliari, Italy; 4 Department of Pharmacology, Dalhousie University, Halifax, NS Canada; 5 Department of Psychiatry, Dalhousie University, Halifax, NS Canada; 6 Department of Clinical Medicine, Aalborg University, Aalborg, Denmark; 7 Psychiatry-Aalborg University Hospital, Aalborg, Denmark; 8 Department of Adult Psychiatry, Poznan University of Medical Sciences, Poznan, Poland; 9 Department of Psychiatric Nursing, Poznan University of Medical Sciences, Poznan, Poland; 10 Department of Neuroscience, Section of Psychiatry, Università Cattolica del Sacro Cuore, Rome, Italy; 11 Fondazione Policlinico Universitario Agostino Gemelli IRCCS, Rome, Italy; 12 Institute of Psychiatric Phenomics and Genomics (IPPG), University Hospital, LMU Munich, Munich, Germany; 13 Department of Psychiatry and Behavioral Sciences, SUNY Upstate Medical University, Syracuse, NY USA; 14 Department of Psychiatry and Psychotherapy, University Medical Center (UMG), Georg-August University Göttingen, Göttingen, Germany; 15 Department of Psychiatry and Behavioral Sciences, Johns Hopkins University, Baltimore, MD USA; 16 Department of Genetic Epidemiology, Central Institute of Mental Health, Mannheim, Germany; 17 International Consortium for Research on Mood & Psychotic Disorders, McLean Hospital, Belmont, MA USA; 18 Department of Psychiatry, Harvard Medical School, Boston, MA USA; 19 Lucio Bini Mood Disorders Centers, Cagliari and Rome, Italy; 20 Department of Psychiatry and Psychotherapy, University Hospital Carl Gustav Carus, Medical Faculty, Technische Universität Dresden, Fetscherstr. 74, 01307 Dresden, Germany

**Keywords:** Bipolar disorder, GSK-3β, Inositol, Virus, Coronavirus

## Abstract

**Background:**

Since its introduction in modern medicine, naturalistic observations emerged about possible uses of lithium treatment for conditions different from recurring affective disorders, for which it is still a first-line treatment option. Some evidence about the antiviral properties of lithium began in the early 1970s, when some reports found a reduction of labial-herpetic recurrences. The present review aims to present most of the pre-clinical and clinical evidence about lithium’s ability to inhibit DNA and RNA viruses, including *Coronaviridae*, as well as the possible pathways and mechanisms involved in such antiviral activity.

**Main body:**

Despite a broad number of in vitro studies, the rationale for the antiviral activity of lithium failed to translate into methodologically sound clinical studies demonstrating its antiviral efficacy. In addition, the tolerability of lithium as an antiviral agent should be addressed. In fact, treatment with lithium requires continuous monitoring of its serum levels in order to prevent acute toxicity and long-term side effects, most notably affecting the kidney and thyroid. Yet lithium reaches heterogeneous but bioequivalent concentrations in different tissues, and the anatomical compartment of the viral infection might underpin a different, lower need for tolerability concerns which need to be addressed.

**Conclusions:**

Lithium presents a clear antiviral activity demonstrated at preclinical level, but that remains to be confirmed in clinical settings. In addition, the pleiotropic mechanisms of action of lithium may provide an insight for its possible use as antiviral agent targeting specific pathways.

## Background

More than 70 years since introduction to routine clinical practice, lithium remains the first-line option for the treatment of bipolar disorder (BD), having the strongest evidence supporting both its acute and long-term efficacy in patients with BD (Yatham et al. 2018). Indications for use of lithium in BD span across different age groups, from children/adolescents (Duffy et al. 2018; Duffy and Grof 2018) to elder populations (Young et al. 2017). In addition to its established clinical efficacy, lithium is associated with a reduction of suicide risk (Tondo and Baldessarini 2018), which is exerted irrespective of its mood-stabilizing properties (Manchia et al. 2013; Sarai and Mekala 2018) and possibly at concentrations as low as those found in drinking water (Barjasteh-Askari et al. 2020). Importantly, lithium contributes to reduction of depressive morbidity, which is predominant in the clinical course of BD (Murru et al. 2017a; Samalin et al. 2016), and is associated with an excess of mortality (Baldessarini et al. 2020).

Despite the well-established efficacy of lithium, its use has declined in the last decades in some parts of the world, partly due to the safety concerns, which require proper therapeutic monitoring, and to strong marketing strategies supporting the use of anticonvulsants and antipsychotics, which however may be less effective (Tondo et al. 2019). Recent evidence shows that the renal toxicity and teratogenic effects of lithium are much less pronounced than initially thought (Fornaro et al. 2020; Nielsen et al. 2018).

### Lithium molecular effects

Lithium has a pleiotropic mechanism of action modulating first, second and third messengers (and their downstream molecular cascades),higher order biological systems (Alda 2015; Quiroz et al. 2004), including the circadian clock rhythm (McCarthy 2019) and the neural plasticity (Alda 2015). Although a detailed review of lithium molecular effects lies outside the scope of this review, some mechanisms might be relevant with regard to its antiviral effects: [a] the inhibition of the phosphatidylinositol signalling pathway via suppression of the inositol-polyphosphate 1-phosphatase (IPPase) and inositol monophosphate phosphatase (IMPase) (Yu and Greenberg 2016), [b] the regulation of autophagy (Motoi et al. 2014), and [c] the inhibition of the glycogen synthase kinase-3, isoform β (GSK-3β) (Quiroz et al. 2004).

Experimental evidence shows that both IMPase and IPPase, which are members of a group of at least four magnesium-dependent phosphomonoesterases, are significantly inhibited at therapeutic serum concentrations of lithium (0.6–1.2 mM/l) (Quiroz et al. 2004). Inositol is the essential substrate for the synthesis of phosphatidylinositol (PI), from which PI(4,5) biphosphate (P2) is produced (Yu and Greenberg 2016). With the receptor-mediated activation of the phospholipase C (PLC), PI(4,5)P2 is cleaved to form inositol-1,4,5-triphosphates (IP3) and 1,2-diacylglycerol (DAG) (Streb et al. 1983). The IP3 can be either recycled to myo-inositol by a series of dephosphorylations catalysed by IPPase and IMPase or, alternatively, can be phosphorylated sequentially to form IP4, IP5, IP6, IP7 and IP8 by a series of kinases, including the inositol polyphosphate multikinase (IPMK) and inositol pentakisphosphate 2-kinase (IPPK) (Balla 2013; Wei et al. 2018; Yu and Greenberg 2016). The depletion of myoinositol determined by the lithium-induced inhibition of IMPase and IPPase, could in turn provoke the dampening of PI signalling with decreased downstream levels of inositol phosphates. Although experimental data suggest that long-term, rather than short-term, lithium exposure can dampen PI signalling (Wei et al. 2018), this molecular effect if of great interest in terms of potential antiviral effects. Indeed, IP6 appears to be a key factor in substantially increasing the viral stability of HIV, an RNA virus, facilitating the accumulation of newly synthesized DNA inside the capsid (Mallery et al. 2018). It is conceivable that decreased levels of IP6 would decrease the ability of HIV of replicating effectively.

Replicated data show that lithium promotes autophagy, the physiological process responsible for the quality control of essential cellular components by purging the cell of old or damaged organelles in several neuropsychiatric conditions (Motoi et al. 2014). The beneficial effect of lithium on autophagy has been demonstrated in several neurodegenerative disorders, including Huntington’s disease (Sarkar et al. 2005) and amyotrophic lateral sclerosis (Fornai et al. 2008) and it appears to be mediated through its modulatory effects on GSK-3β and IMPase (Motoi et al. 2014). As several DNA and RNA viruses are able to inhibit the autophagy pathway to increase their survival (Mehrbod et al. 2019), this molecular effect of lithium may decrease the chance of the viruses’ survival.

Finally, the relevant inhibitory action of lithium on GSK-3ß, a serine-threonine kinase that influences more than one hundred substrates modulating cell survival, gene expression and microtubule formation, appears relevant (Alda 2015; Quiroz et al. 2014). In fact, the inhibition of GSK-3ß during the later stages of infection with Dengue virus-2 (DENV-2), an RNA virus, resulted in a reduction of viral titres in hepatocarcinoma cell (Huh-7) and Vero cell lines (Cuartas-López and Gallego-Gómez 2020). In addition, lithium-induced inhibition of GSK-3ß led to a significantly more decreased production of chronic hepatitis C virus (HCV) viral particles in treated vs. untreated human hepatoma cell lines (Sarhan et al. 2017).

### Lithium and immune system

Immune dysfunction seems to plays a key role in the onset and progression of BD in a substantial proportion of individuals (Rosenblat 2019). Lithium has long been recognized as an immune modulating drug (Rybakowski 1999), with both anti-inflammatory (e.g., suppression of cyclooxygenase-2 expression, inhibition of interleukin (IL)-1β and tumour necrosis factor-α production, and enhancement of IL-2 and IL-10 synthesis) and pro-inflammatory (e.g., induction of IL-4, IL-6 and other pro-inflammatory cytokines synthesis) action (Nassar and Azab 2014). In the long term, however, the use of lithium has been significantly associated with a normalization of cytokine levels, balancing the disruptions observed in BD patients (Van Den Ameele et al. 2016). Therefore, lithium exerts a combined action that involves multiple pathways. This discloses different potential applications of lithium which remain largely unexplored (Chiu et al. 2013).

## Aim of the review

The recent pandemic of the 2019 novel coronavirus (SARS-CoV-2) causing the coronavirus disease (CoViD-19) has emphasized the need for any effective treatment, given the few therapeutic options available (Guan et al. 2020). The only therapeutic strategies currently available are those applied in intensive care units, i.e. using anti-inflammatory agents and anticoagulants to prevent the respiratory insufficiency and the vasculitis. Some antiviral agents, namely the recently FDA-approved remdesivir, help the viral elimination although conclusive evidence on its efficacy is still lacking (Grein et al. 2020; Ledford 2020). In addition, almost all countries have suggested or disposed lockdown measures able to tackle down the spread of the infection such as social distancing, quarantine, and isolation (Baden and Rubin 2020), all presenting major challenges and limitations (Niud and Xu 2020). The development of a vaccine represents the ideal therapeutic approach to the CoViD-19 pandemic, but despite some progress it can be still lengthy. At the moment, symptomatic approaches to the infection and its complications, combined with re-purposing of therapeutic options already available for other conditions, are two main areas of action against this viral pandemic (Lu 2020).

In this context, we aim to review the preclinical and clinical evidence on the antiviral effects of lithium, offering a perspective for its potential use in clinical settings. For the sake of clarity, in each section we will present first the effect of lithium on DNA viruses and then on RNA viruses, including the family of *Coronaviridae*.

We performed a broad literature search including the keywords “lithium” and “antiviral”, “viral”, alone and with “*” wildcard, in order to screen for the widest result output. We cross-checked references for articles of interest. We excluded from our results opinion articles, editorials or reviews, and articles which were not written in English language.

## Main text

### Preclinical evidence

#### DNA viruses

The first report on lithium antiviral effects dates back to 1970, when Neurath et al. (1970) showed that lithium iodide disrupted the viral capsid of adenovirus type 7 (Neurath et al. 1970). Subsequently, lithium iodide’s ability to degrade the nucleocapsid was observed also in the Herpes simplex virus (HSV) (McCombs and Williams 1973). In addition, lithium was first shown to inhibit the replication of type 1 and type 2 HSV at concentrations of 5 mM/l and of the pseudorabies and vaccinia viruses (Skinner et al. 1980). This effect extended also to pseudorabies and vaccinia virus (Skinner et al. 1980). Further support was provided for the in vitro antiviral activity of lithium on HSV in Vero cells and rabbit (Trousdale et al. 1984). However, the same authors were unable to detect a reduction in the reactivation of latent infection in rabbits (Trousdale et al. 1984).

Cernescu et al. (1988) observed a reduction in virus replication in human embryo fibroblasts cultures infected with measles or HSV when pre-treated with lithium chloride at concentrations of 1–10 mM/l (Cernescu et al. 1988). The maximum effect was obtained by a 1-h treatment with 10 mM/l lithium chloride, preceding viral infection by 19–24 h (Cernescu et al. 1988). Further, they showed that intermittent treatment with 10 mM lithium chloride of cultures persistently infected with measles or HSV obtained from human myeloid K-562 cell line showed a reduction in the extracellular virus yield (Cernescu et al. 1988). Of interest, lithium not only reduced vital replication, but also restored the synthesis of almost all host-cell proteins, including fibronectin, type IV collagen, thrombospondin (TSP) and proteoglycans, which is typically suppressed by HSV (Ziaie and Kefalides 1989). Again, lithium was more effective at the higher concentration (30 mM/l) and when the compound was added to the culture at the time of infection rather than after adsorption of HSV (Ziaie and Kefalides 1989).

One proposed possible mechanism of lithium inhibiting effect on HSV DNA synthesis is the displacement of potassium from a potassium-dependent biochemical event or through other physiological change following the loss of cellular potassium (Hartley et al. 1993). In addition, it is plausible that lithium also directly inhibits viral replication. Indeed, lithium chloride at 30 mM suppressed the synthesis of viral polypeptides, whereas the synthesis of host proteins was maintained. In particular, the mRNAs for viral proteins, including the DNA polymerase, were nearly undetectable when lithium was added with the virus to the endothelial cell cultures infected with HSV-1 (Ziaie et al. 1994).

Some studies focused on the effects of lithium on virus of the Parvoviridae family. Chen et al. (2015) reported on the inhibition of porcine parvovirus (PPV) replication in swine testis (ST) cells by lithium chloride in a dose-dependent fashion, with statistically significant effects observable already at 5 mM/l (Chen et al. 2015). As for other viruses, the antiviral effect of lithium chloride occurred in the early phase of PPV replication (Chen et al. 2015). In addition, Zhout et al. (2015) showed that lithium not only suppressed the synthesis of viral DNA and proteins of canine parvovirus in a dose-dependent manner, but also inhibited viral entry into feline kidney cells cultures (Zhou et al. 2015).

#### RNA viruses

A series of studies have explored the effects of lithium antiviral activity on RNA viruses both in cellular and animal models. Gallicchio et al. (1993) explored the hypothesis that lithium treatment might decrease the severity of murine acquired immune deficiency syndrome (MAIDS) induced by the murine leukaemia retrovirus (Gallicchio et al. 1993). Lithium-treated animals (1 mM/l) demonstrated a marked reduction in the development of lymphadenopathy and splenomegaly suggesting a potential role of lithium in the pathophysiological processes associated with retroviral infections (Gallicchio et al. 1993).

These antiviral effects appear to extend to other RNA viruses such as those pertaining to the family of *Coronaviridae*. Harrison et al. (2007) tested the effect of lithium chloride on the replication of avian coronavirus infectious bronchitis virus (IBV) in cell culture using two model cell types: Vero cells, an African Green monkey kidney-derived epithelial cell line, and DF-1 cells, an immortalized chicken embryo fibroblast cell line (Harrison et al. 2007). When treated with a range of lithium chloride concentrations (0, 5, 10, 25 or 50 mM/l), IBV RNA and protein levels, as well as viral progeny production were reduced in a dose-dependent manner in both cell types, with data indicating that the inhibition was determined by a cellular, by inhibiting RNA synthesis, rather than a virucidal effect (Harrison et al. 2007), an effect also confirmed in a subsequent study by Li et al. (2009) (Li et al. 2009). Furthermore, in *Vero* cells, lithium chloride showed effectiveness in suppressing infection of the porcine epidemic diarrhoea virus by inhibiting of the virus entry, replication and apoptosis (Li et al. 2018). In type II porcine reproductive and respiratory syndrome virus lithium chloride reduced RNA production and protein transduction (Cui et al. 2015). Furthermore, lithium chloride at concentrations of 10–60 mM significantly inhibited viral replication of porcine deltacoronavirus (PDCoV) in porcine kidney cells (LLC-PK1) compared to mock-treated cells (Zhai et al. 2019). The antiviral effects of lithium chloride occurred at the early stage of PDCoV replication, and was associated to the inhibition of the PDCoV-induced apoptosis in LLC-PK1 cells (Zhai et al. 2019). Finally, lithium chloride showed in vitro ability to limit both early and late stages of infection and to inhibit apoptosis in another porcine coronavirus causing transmissible gastroenteritis (Ren et al. 2011).

In another study, lithium chloride inhibited the replication of the foot-and-mouth disease virus (FMDV) (Zhao et al. 2017). The viral titres of FMDV decreased in a dose-dependent manner in cells cultures, although it did not affect FMDV attachment stage and entry stage in the course of its life cycle (Zhao et al. 2017). Finally, two studies confirmed the inhibitory effects of lithium on viral replication in other RNA viruses such a feline calicivirus (FCV) (Wu et al. 2015), and mammalian orthoreoviruses (Chen et al. 2016). Wu et al. (2015) showed that lithium chloride effectively suppressed the replication of FCV strain F9 in Crandell-Reese feline kidney (CRFK) cells in a dose-dependent manner and inhibited the virus-induced cytopathic effect (Wu et al. 2015). The dose-dependent inhibition of viral replication was observed also in reovirus infected Vero cells (Chen et al. 2016).

### Clinical evidence

#### DNA viruses

Early observations reported that depressed and bipolar depressed patients presented increased antibodies titres to HSV (Lycke et al. 1974). Few years later, between 1979 and 1983, some cases were published reporting on the possible antiviral effect of lithium in humans, with the observed remission of labial (HSV1) herpes in 3 lithium carbonate-treated affective patients (Gillis 1983; Lieb 1979). In these cases, lithium was initiated for a chronic recurring affective disorder in patients with personal history for frequent labial herpes manifestations, and it reduced or interrupted herpetic recurrences. Furthermore, at lithium discontinuation, labial herpes recurred with previous frequency.

These serendipitous findings awoke interest on the possible immune-modulatory and/or antiviral action of lithium. A retrospective study followed (Amsterdam et al. 1990), including a total of 263 patients. Of them, 177 subjects received lithium carbonate prophylaxis, while a comparison group of 59 subjects received antidepressant monotherapy for a major affective disorder. Overall, 90 out of 236 subjects reported the presence of recurrent labial herpes infections, 63/177 (36%) on lithium and 27/59 (46%) on antidepressants with not statistically significant difference in the rates. However, the mean pre-treatment recurrence rate for labial herpes infections (1.6 ± 2.6/year) significantly decreased during treatment (0.8 + 1.8/year, p < 0.001). In contrast, the same recurrence rates showed no significant changes in antidepressant-treated patients (Amsterdam et al. 1990). Of note, the reduction of HSV recurrences was higher in patients with lithium concentrations ≥ 0.65 mmol/l than in those with lower concentration (respectively, 70% vs. 54%) and with erythrocyte lithium levels ≥ 0.35 mmol/l than patients with lower concentrations (respectively, 81% vs. 49%) (Rybakowski and Amsterdam 1991). Afterwards, the Polish arm (28 patients) of the previous study was followed-up in an uncontrolled prospective report to further study the prophylactic effect of lithium carbonate against HSV recurrences (Rybakowski et al. 1996). The observed reduction of HSV recurrences did not correlate with lithium concentrations in serum or erythrocytes.

Importantly, lithium concentration in plasma is considerably lower than the concentrations showing anti-viral properties in in vitro trials, but lithium concentration in saliva is considerably higher than in plasma, and both concentrations show bioequivalence (Murru et al. 2017b), so that a direct and topic effect on labial mucosae is hypothesized. The observation that lithium may heterogeneously accumulate in different tissues prompted a randomized double blind, placebo-controlled trial on the use of topic 8% lithium succinate ointment in 73 patients with recurring genital (HSV2) herpes (Skinner 1983). The ointment was applied 4 times a day for 7 days, swabs from lesions obtained at day 4 or 5 after onset of lesions, and a quantitative measure of HSV2 was performed. The median duration of pain/discomfort was reduced in lithium-treated patients from 7 to 4 days (p < 0.05), while time to full healing was decreased from 8 days in the placebo arm to 7 days in the active drug arm. HSV2 excretion at day 4 or 5 was present in 11/20 (55%) placebo-treated compared with 5/37 (14%) lithium-treated patients, and virus concentration in lithium group was reduced by a 30-fold as compared to the concentration in the placebo arm (p < 0.05). Lithium succinate ointment showed good active tolerability, with no side effects reported (Skinner 1983).

Positive results were also obtained in an uncontrolled study conducted on a sample of 42 Polish patients (38% female) with recurrent labial (HSV1) herpes with illness duration of 1–25 years and frequency of recurrences varying from very frequent (1/month) to rare (1 recurrence every 7 months or more) (Rybakowski et al. 1991). Lithium succinate 8% ointment was the tested drug, applied topically within 1–4 days of lesion onset, 2–7 times days the first 3 days and 1–2 times per day thereafter. All patients achieved full recovery in 2–7 days (mean 4 days), with subjective complaints alleviated after the first 1–3 applications. During the follow-up (ranging 4–12 months), when relapse was observed (6/42), lesions never occurred in the same location of the lesion at study entry (Rybakowski et al. 1991).

Oral lithium carbonate treatment was tested as a prophylactic recurrence treatment of HSV-2 in two randomized, placebo-controlled trials. In the first one (Amsterdam et al. 1991), 10 women with recurrent genital HSV infection entered oral lithium for 12 months, and were followed for a total duration of 18 months. During the active treatment phase, average daily lithium doses were 587 + 49 mg and average plasma levels were 0.51 mmol/l. Patients in the active arm of the study showed a trend towards average monthly reduction in number and duration of herpetic lesions, maximum symptom severity and clinical severity.

In the second randomized controlled trial (Amsterdam et al. 1996), 11 patients (9 women), aged 38 ± 11 years (range 28–65) and with a personal history of HSV-2 infection with four or more recurrences were randomly assigned to lithium (n = 6) or placebo (n = 5) for at least five months. The mean number of manifestations in the year before study entry was 12 ± 8 (range 4–30), each episode lasting 12 ± 8 days. Mean lithium daily dosage was 437 ± 185 mg (range 150–900 mg/day), with serum concentration of 0.56 ± 0.20 mmol/l. Differences between study arms were statistically nonsignificant and pointing to an overall attenuation of HSV-2 in lithium-treated patients, while placebo-treated patients showed a worsening in 3 of 4 infection clinical outcomes. Last, a case reports an adolescent female BD patient, with a history of chronic active Epstein Barr virus infection and recurrent acute pancreatitis, who achieved apparent control of the viral infection with lithium monotherapy (Pavuluri and Smith 1996).

#### RNA viruses

Early reports on lithium antiviral properties reported minimal or no effect on RNA viruses, apart from an anecdotic observation of reduced symptoms of common cold and influenza in a retrospective study focused on anti-herpetic action of lithium (Rybakowski and Amsterdam 1991).

A proof-of-concept study (Puertas et al. 2014), was conducted on 9 patients affected by human immunodeficiency virus-1 (HIV) previously recruited in a trial investigating the possible neurocognitive protecting effect of rivastigmine, compared with lithium carbonate (Muñoz-Moreno et al. 2017). The aim of the proof-of-concept study was to study the effect of lithium on HIV-1 expression and reservoir size in the CD4þ T cells of virologically suppressed patients. Mean time from HIV-1 diagnosis was 10.7 ± 6.5 years, and the mean time of sustained virological suppression was 5.3 ± 3.4 years. Patients started with an initial lithium carbonate dose of 400 mg/day and lithium blood levels of 0.4–0.8 mmol/l. At week 2, cell-associated HIV-1 RNA transcripts decreased in 5 of 6 patients. At week 4, the reduction in viral transcription levels was of 40%. Of note, viral transcription later increased so that at week 12 of treatment all patients recovered their initial transcription pattern. Viremia decreased from 67% at baseline to 44% immediately after beginning lithium, possibly by activation of the ß-catenin signalling, but rose to 87% at week 12. These data reflected the patterns of expression of HIV-1 in circulating CD4þ T cells. The proportion of circulating CD4þ T cells harbouring proviral DNA was also measured. At baseline, HIV-1 copies were 1173 per million CD4þ T cells (interquartile range: 388–2343) and at week 4, they dropped to 582 copies (373–1606), a significant reduction in the size of the proviral reservoir in CD4þ T cells (19% median decrease, p = 0.03), later lost at week 12 (Puertas et al. 2014).

A retrospective study conducted on a sample of 236 affective disorder patients 177 taking lithium carbonate and 59 taking antidepressants (tricyclics, monoamine oxidase inhibitors or fluoxetine) on a chronic basis, investigated the possible effect of psychotropic drugs in reducing the reported yearly recurrence rate of flu-like infections (Amsterdam et al. 1998). Results of this preliminary report showed a statistically significant reduction in mean yearly rates of flu-like infections in lithium- (pre-treatment 1.48 ± 1.13 vs. post-treatment 1.14 ± 1.20, p < 0.001), but not antidepressant-treated patients.

## Discussion

In this narrative review we have summarized the studies on preclinical and clinical efficacy of lithium as antiviral agent. A series of considerations should be made with regard to each set of findings. In general, early pre-clinical studies converge on the ability of lithium to inhibit viral replication. This effect has been extensively found on a wide group of DNA viruses, mostly of the *Herpesviridae* family, including the HSV-1, HSV-2, Epstein-Barr virus, Cytomegalovirus, and adenovirus, and it extends to other RNA viruses, among which are the reovirus, HVC, avian leucosis virus, and different viruses of the *Coronaviridae* family (Nowak and Walkowiak 2020).

However, in most cases findings from in vitro studies lack supportive clinical evidence. Indeed, the available clinical evidence appears scant and generally of low quality, as it is limited to case series and retrospective studies. In fact, only two randomized controlled studies have been published, whose results weakened by small sample sizes and consequent inadequate statistical power. It is possible that the insufficient proper clinical evidence of the antiviral effects of lithium is a reflection of its status as a drug without strong marketing support. Indeed, after two seminal randomized clinical trials (Amsterdam et al. 1991, 1996), there have been no further investigation of this important property of lithium, although supported by some preclinical evidence summarized in this review. The gap in the knowledge of lithium clinical spectrum of efficacy beyond the well-known effects in the prevention of mood-disorders recurrence has substantial implications in the context of the current pandemic of the CoViD-19.

The pandemic has first expanded from the Wuhan region in China, and has quickly spread to Europe and to the rest of the world, that, if not still struggling for an adequate control of the pandemic (Guan et al. 2020), remains in alert for future, possibly seasonal manifestation of the outbreak (Kissler et al. 2020). Whilst the development of a CoViD-19 vaccine is the desired advancement for the control of the pandemic, the growing prevalence of symptomatic CoViD-19 patients calls for a broadening of possible therapeutic options. The development of treatments for CoViD-19 is feasible either by testing known or unknown existing broad-spectrum antiviral treatments (Baden and Rubin 2020), like the recently FDA-approved remdesivir (Grein et al. 2020). Although, the most reasonable step hast to be the development of new, specific compounds, would require a considerable amount of time for the entire drug-development pipeline. Alternatively, repurposing of some drugs marketed with other clinical indications than antiviral, but showing efficacy against the SARS-CoV-2 could be a viable option. Lithium may have a relevant role in this scenario.

As summarized by our group and by others (Nowak and Walkowiak 2020) the antiviral effects of lithium extend to several components of the *Coronaviridae* family. In addition, further support, although indirect, for the role of lithium appears to come from advanced analysis combining structure-assisted drug design, virtual drug screening and high-throughput screening to repurpose existing drugs to target SARS-CoV-2, and particularly its main protease M^pro^, which is an essential component for viral replication (Jin et al. 2020). Indeed, this study showed that ebselen, a lithium mimetic agent (Singh et al. 2013), determined an enzymatic inhibition of the protease M^pro^ activity, a finding further substantiated the in vitro observation of a strong antiviral effect at a concentration of 10 μM in CoViD-19 virus infected Vero cells (Jin et al. 2020). This appears relevant given that ebselen shares a distinct molecular mechanism with lithium, namely the inhibition of IMPase (with consequent dampening of PI signalling) which induces lithium-like effects on mouse behaviour, reversed by inositol (Pisanu et al. 2016; Singh et al. 2013).

However, the repurposing of lithium may pose specific problems. Among them, regulatory requirements which underwent an attempt to harmonize between national and federal medication agencies dating as back as 2007 (Mariz et al. 2016). Furthermore, the need for medical plausibility as well as for a solid scientific rationale, both at pre-clinical and clinical level, needs to combine with complex safety/surveillance monitoring, which may benefit from large, structured and integrated nationwide data sources (Crisafulli et al. 2019). Lithium’s safety and tolerability profile is a major concern in clinical practice, and its narrow therapeutic window needs accurate monitoring to optimize its effectiveness as well as increase treatment adherence (Nolen et al. 2019). Experimental data show that viral inhibition starts to occur at very high concentrations of lithium, typically close to the toxicity threshold reported in human pharmacokinetic studies (1.0–1.2 mM/l). However, it should be noted that, although the in vitro antiviral effects are more prominent at higher concentrations of lithium, some level of activity is detectable even at lower doses. In addition, it is conceivable that, as previously reported for saliva, specific anatomic compartment can have higher concentrations of lithium than those detectable in serum. Thus, as demonstrated for labial HSV, the normal therapeutic levels could be sufficient to obtain some degree of antiviral efficacy. Furthermore, specific dosage regimens, such as that including the evening loading of lithium, might be more effective in this regard and should be considered in eventual clinical studies.

On the other hand, considering the viral direct liver damage CoViD-19 (Zhang et al. 2020) and the possible hepatotoxicity to either antiviral drugs, i.e. remdesivir, or the underlying disease (Grein et al. 2020), lithium’s null effect on hepatic function could prove useful. In fact, despite lithium treatment for CoViD-19 in the general population seems unlikely, when tailoring a treatment plan for CoViD-19in patients affected by BD, the awareness of lithium’s antiviral effect could optimize the inevitable polypharmacy. Patients affected by BD present increased rated of cardiovascular disease, metabolic syndrome, diabetes, overweight/obesity, hypertension and smoking status compared to the general population (Vancampfort et al. 2015; Vancampfort et al. 2013). Such comorbidities associate with a worse CoViD-19 outcome, both directly with increased in-hospital deaths due complications (Mehra et al. 2020) and indirectly due to worst outcomes when subjected to mechanical ventilation (Martínez-Alés et al. 2020).

We believe that the findings summarized should motivate two types of investigations to build a rationale for clinical investigations on its effects on *Coronaviridae*. One set of evidence should come from in vitro studies directly testing the antiviral effect of lithium in cell cultures infected with the SARS-CoV-2. This type of tests may require also a relatively long time, whereas naturalistic and simple investigations could aim at identifying effects on the CoViD-19 on infected lithium-treated and untreated subjects.

One final remark should be made concerning the methodology applied in this work. As described, we performed a narrative review that lacks the proper systematic approach needed for a qualitative (or quantitative) synthesis of the literature. However, it should be noted that, even if we did not apply specific inclusion or exclusion criteria, our search was quite thorough and included also studies tracked via accurate reference checking.

## Conclusions

In conclusion, lithium has clear antiviral activity that is demonstrated at preclinical level but remains to be established in clinical settings. A direct inhibitory effect of lithium on viruses of the *Coronaviridae* family and on the SARS-CoV-2 in particular remains a key, and yet unanswered research question.

## Data Availability

The data will not be shared or made publicly available.

## Abbreviations

CoViD-19: Coronavirus disease
CRFK: Crandell–Reese feline kidney
DAG: 1,2-Diacylglycerol
DENV-2: Dengue virus-2
FCV: Feline calicivirus
FMDV: Foot-and-mouth disease virus
GSK-3β: Glycogen synthase GSK-3β kinase-3, isoform β
HCV: Chronic hepatitis C virus
HIV: Human immunodeficiency virus-1
HSV: Herpes simplex Virus
IBV: Infectious bronchitis virus
IL: interleukin
IP3: Inositol-1,4,5-triphosphates
IMPase: Inositol monophosphate phosphatase
IPMK: Inositol polyphosphate multikinase
IPPase: Inositol-polyphosphate 1-phosphatase
IPPK: Inositol pentakisphosphate 2-kinase
P2: Biphosphate
PDCoV: Porcine deltacoronavirus
PI: Phosphatidylinositol
PI(4,5): Phosphatidylinositol biphosphate
PLC: Phospholipase C
PPV: Porcine parvovirus
ST: Swine testis
TSP: Thrombospondin
